# Tumor exome sequencing and copy number alterations reveal potential predictors of intrinsic resistance to multi-targeted tyrosine kinase inhibitors

**DOI:** 10.18632/oncotarget.22914

**Published:** 2017-12-04

**Authors:** Nancy K. Gillis, Daniel M. Rotroff, Tania E. Mesa, Jiqiang Yao, Zhihua Chen, Michael A. Carulli, Sean J. Yoder, Christine M. Walko, Jamie K. Teer, Howard L. McLeod

**Affiliations:** ^1^ DeBartolo Family Personalized Medicine Institute, H. Lee Moffitt Cancer Center and Research Institute, Tampa, FL, USA; ^2^ Department of Cancer Epidemiology, H. Lee Moffitt Cancer Center and Research Institute, Tampa, FL, USA; ^3^ Center for Pharmacogenomics and Individualized Therapy Eshelman School of Pharmacy, University of North Carolina, Chapel Hill, NC, USA; ^4^ Bioinformatics Research Center, North Carolina State University, Raleigh, NC, USA; ^5^ Molecular Genomics Core, H. Lee Moffitt Cancer Center and Research Institute, Tampa, FL, USA; ^6^ Cancer Informatics Core, H. Lee Moffitt Cancer Center and Research Institute, Tampa, FL, USA; ^7^ College of Pharmacy, University of South Florida, Tampa, FL, USA; ^8^ Department of Biostatistics and Bioinformatics, H. Lee Moffitt Cancer Center and Research Institute, Tampa, FL, USA

**Keywords:** tyrosine kinase inhibitors, resistance, somatic genetics, intrinsic, copy number

## Abstract

Multi-targeted tyrosine kinase inhibitors (TKIs) have broad efficacy and similar FDA-approved indications, suggesting shared molecular drug targets across cancer types. Irrespective of tumor type, 20-30% of patients treated with multi-targeted TKIs demonstrate intrinsic resistance, with progressive disease as a best response. We conducted a retrospective cohort study to identify tumor (somatic) point mutations, insertion/deletions, and copy number alterations (CNA) associated with intrinsic resistance to multi-targeted TKIs. Using a candidate gene approach (n=243), tumor next-generation sequencing and CNA data was associated with resistant and non-resistant outcomes. Resistant individuals (n=11) more commonly harbored somatic point mutations in *NTRK1*, *KDR*, *TGFBR2*, and *PTPN11* and CNA in *CDK4*, *CDKN2B*, and *ERBB2* compared to non-resistant (n=26, p<0.01). Using a random forest classification model for variable reduction and a decision tree classification model, we were able to differentiate intrinsically resistant from non-resistant patients. CNA in *CDK4* and *CDKN2B* were the most important analytical features, implicating the cyclin D pathway as a potentially important factor in resistance to multi-targeted TKIs. Replication of these results in a larger, independent patient cohort has potential to inform personalized prescribing of these widely utilized agents.

## INTRODUCTION

Increasing evidence supports the classification of tumors based on genetic and molecular characteristics rather than site of origin, which has translated into the ability to predict drug response utilizing tumor genetics rather than tumor type (i.e., histology or site of origin) in some instances [1]. For example, using multiple genetic platforms, The Cancer Genome Atlas Research Network defined four molecular subtypes of breast cancer (Luminal A, Luminal B, HER2-enriched, and Basal-like) and found that one subtype (Basal-like) is more similar to serous ovarian cancer than to other breast cancers [2]. Further analysis showed that, due to their molecular similarities, Basal-like breast cancers and serous ovarian cancers are likely susceptible to similar targeted treatments. In another multiplatform analysis of twelve tissue-defined cancer types, eleven major subtypes were identified, with only five subtypes corresponding to their tissue of origin, and the remaining six subtypes being shared by distinct cancer types (e.g., lung squamous, head and neck, and some bladder cancers fell into a single subtype) [3]. Studies such as these have influenced clinical cancer drug development. The classification and treatment of tumors based on molecular alterations is currently being studied through the use of basket trials, such as NCI-MATCH and NCI-MPACT, which randomize patients to an individualized targeted therapy arm or a non-pathway-specific arm independent of tumor histology [4]. We hypothesize that genetic alterations within tumors, regardless of site of origin or histology, can be used as a biomarker of response to targeted anticancer therapies.

The broad efficacy of multi-targeted tyrosine kinase inhibitors (TKIs) across tumor types suggests similarities in the genetics of the tumors they are used to treat. The multi-targeted TKIs (i.e., axitinib, cabozantinib, pazopanib, regorafenib, sorafenib, sunitinib, and vandetanib) currently have FDA-approved indications in seven histological tumor types, and are used off-label in additional solid and hematologic malignancies. While the clinical trials of these agents demonstrated overall efficacy, a substantial number of individuals never responded to therapy. This is a clinical challenge for most targeted anticancer agents [5]. In the pivotal phase III clinical trials that led to approval of each of the multi-targeted TKIs, approximately 20 to 30% of patients showed a best response of progressive disease, demonstrating intrinsic resistance [6–13]. In an exploratory study (n=262) examining intrinsic resistance to multi-targeted TKIs across tumor types, we observed an intrinsic resistance rate of 21%, consistent with that seen in clinical trials (unpublished data). No patient demographic or clinical factors (e.g., tumor type or drug received) were associated with intrinsic resistance. This supports the hypothesis that genetic factors may be important in predicting intrinsic resistance to the multi-targeted TKIs. We conducted a retrospective case-control study using a candidate gene approach to identify somatic point mutations, insertions/deletions, and copy number alterations (CNAs) associated with resistance to multi-targeted TKIs across tumor types.

## RESULTS

### Patient population and phenotypes

**Figure 1:**
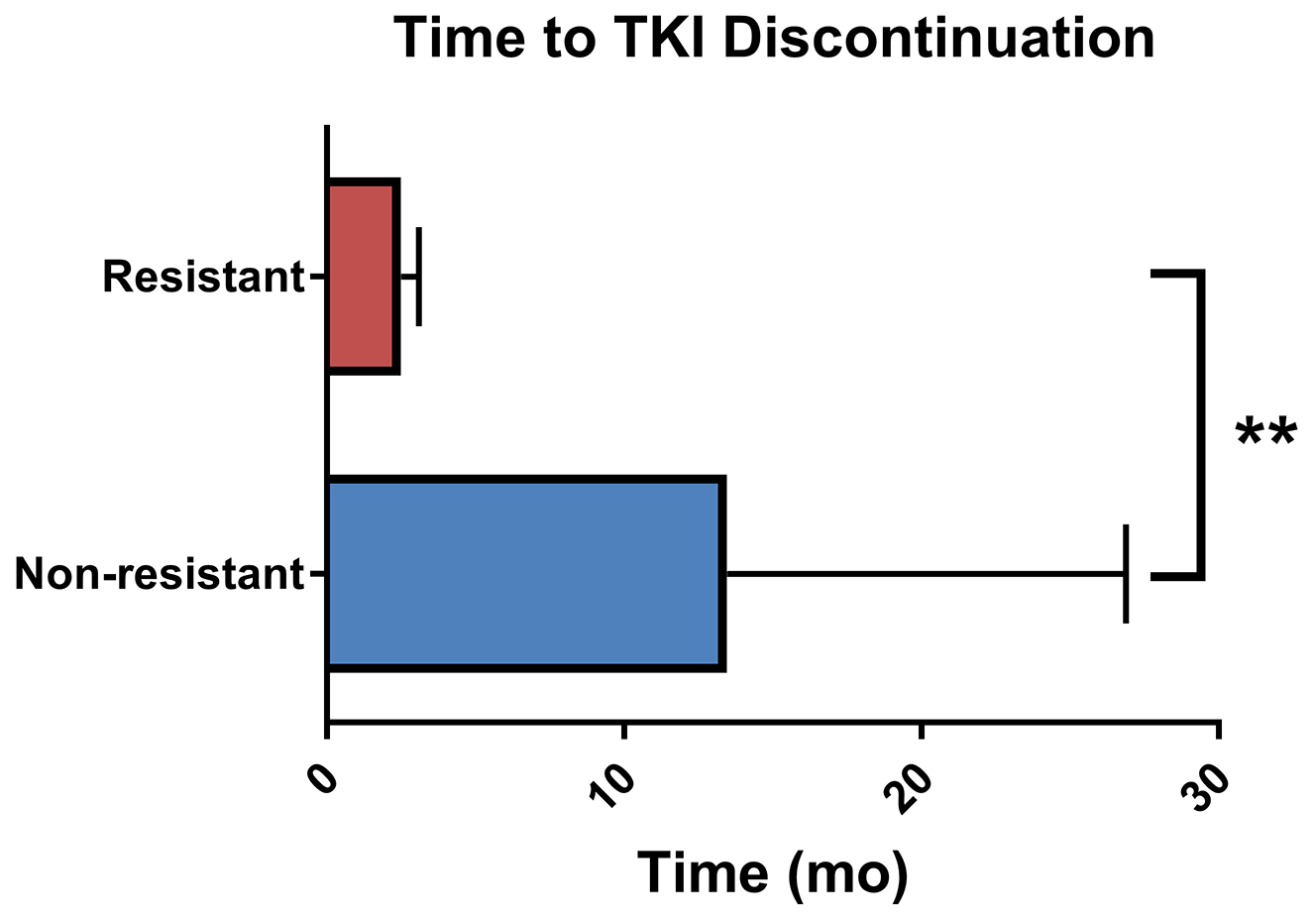
Time to multi-targeted tyrosine kinase inhibitor discontinuation Data represents mean and standard deviation. ^**^Mann-Whitney U p-value < 0.0001.

A total of 50 unique patients were included in this study (Table 1). The average age was 60 years old and the majority of individuals were white (88%) non-Hispanic (90%) males (72%). The most common tumor types being treated were sarcoma (48%) and renal cell carcinoma (40%), as would be expected, and the majority of patients received pazopanib (38%), followed by sorafenib (30%) and sunitinib (28%). One patient received regorafenib and one axitinib. Of the 50 patients included, 11 (22%) were classified as resistant, 26 (52%) as non-resistant, and 13 had unclassifiable responses. This corresponded to an overall resistance rate of 30.5% (11/36) observed in our cohort. After correcting for multiple comparisons using false discovery rate (FDR), there were no statistically significant differences in the demographic and clinical characteristics of resistant and non-resistant patients. Resistant patients discontinued multi-targeted TKIs significantly sooner than non-resistant patients did (U = 14.5, p <0.0001, Figure 1).

**Table 1:** Patient demographics (n = 49) Demographics are broken down by phenotype for individuals who underwent next-generation sequencing (n=37) and copy number alteration analysis (n=29)

		Next-generation sequencing			Copy number alteration		
Characteristic	All patients(n = 50)	Resistant(n = 11)	Non-resistant(n = 26)	P-value(FDR) *^#^*	Resistant(n = 8)	Non-resistant(n = 21)	P-value(FDR) *^#^*
Age^*^				0.19			0.06
Mean ± SD	60.2 ± 12	55.2 ± 13.1	61.2 ± 12.6	(0.39)	52.9 ± 13.9	63.1 ± 11.3	(0.09)
Median	62	61	62.0		58.5	66	
Range	34 – 80	36 – 70	34 – 80		36 – 68	39 – 80	
**Sex**				0.05			0.03
Male	36 (72)	7 (63.6)	24 (92.3)	(0.15)	4 (50)	19 (90.5)	(0.07)
Female	14 (28)	4 (36.3)	2 (7.7)		4 (50)	2 (9.5)	
**Race**				0.66			0.27
White	44 (88)	10 (90.9)	24 (92.3)	(0.80)	7 (87.5)	21 (100)	(0.33)
Black	3 (6)	1 (9.1)	0		1 (12.5)	0	
Asian	2 (4)	0	1 (3.8)		0	0	
Unknown	1 (2)	0	1 (3.8)		0	0	
**Ethnicity**				1.0 (1.0)			0.48
Hispanic	5 (10)	1 (9.1)	3 (11.5)		1 (12.5)	1 (4.8)	(0.48)
Non-Hispanic	45 (90)	10 (90.9)	23 (88.5)		7 (87.5)	20 (95.2)	
**Cancer type**				0.03			0.007
Sarcoma	24 (48)	6 (54.5)	9 (34.6)	(0.15)	4 (50)	5 (23.8)	(0.04)
Renal cell carcinoma	20 (40)	2 (18.2)	16 (61.5)		1 (12.5)	15 (71.4)	
Hepatic	3 (6)	1 (9.1)	1 (3.8)		1 (12.5)	1 (4.8)	
Breast	1 (2)	1 (9.1)	0		1 (12.5)	0	
Colorectal	1 (2)	1 (9.1)	0		1 (12.5)	0	
Melanoma	1 (2)	0	0		0	0	
**Multi-targeted TKI**				0.44			0.03
Pazopanib	19 (38)	5 (45.5)	7 (26.9)	(0.66)	4 (50)	4 (19.0)	(0.07)
Sorafenib	15 (30)	3 (27.3)	10 (38.5)		3 (37.5)	9 (42.9)	
Sunitinib	14 (28)	2 (18.2)	8 (30.8)		0	8 (38.1)	
Axitinib	1 (2)	0	1 (3.8)		0	0	
Regorafenib	1 (2)	1 (9.1)	0		1 (12.5)	0	

^*^ Age represents the age at multi-targeted TKI initiation.

*^#^*P-value for continuous variables represents the logistic regression p-value and categorical data was compared between resistant and non-resistant individuals using Fisher’s exact test. FDR represents the FDR-corrected p-value.

### Next-generation sequencing: quality control

A total of 24 samples underwent targeted exome sequencing under the TCC protocol. The target region was 1,321 genes covering 3.8 Mb. The median number of reads aligned per sample was 15,283,830. Median read depth of coverage was 141x. A median of 93.7% of coding bases were covered at least 10x across samples. For the 25 tumor samples that underwent WES, an average coverage of 151x (95% CI 140 - 163) per base was achieved. The average total number of reads per sample was 1.83 × 10^8^ (95% CI 1.75 × 10^8^ - 1.92 × 10^8^) or 91.5 million paired-end reads, with an average of 20% duplicate reads and 98% of reads mapped to the human reference genome. After removing duplicates, paired-end reads were properly paired overall (average per sample 94%). An average of 74,349 variants (point mutations and insertion/deletions) were detected per sample, of which 22,993 (31%) were within coding regions of the genome, and 11,590 (16%) were non-synonymous variants. There were five genes (*CDK12*, *FGFR4*, *MLL2*, *LRP1B*, and *ARAF*) with significant variation by sequencing method (FDR-corrected p-values all <0.005), warranting exclusion from further analyses. Four genes (*BRCA2, CDK12, MLL2,* and *VHL*) were differentially mutated (p-value < 0.05) between renal cell carcinoma patients and sarcoma patients; however, these differences were not significant when correcting for multiple comparisons (FDR q-values >0.5).

### Next-generation sequencing: genetic associations

**Figure 2:**
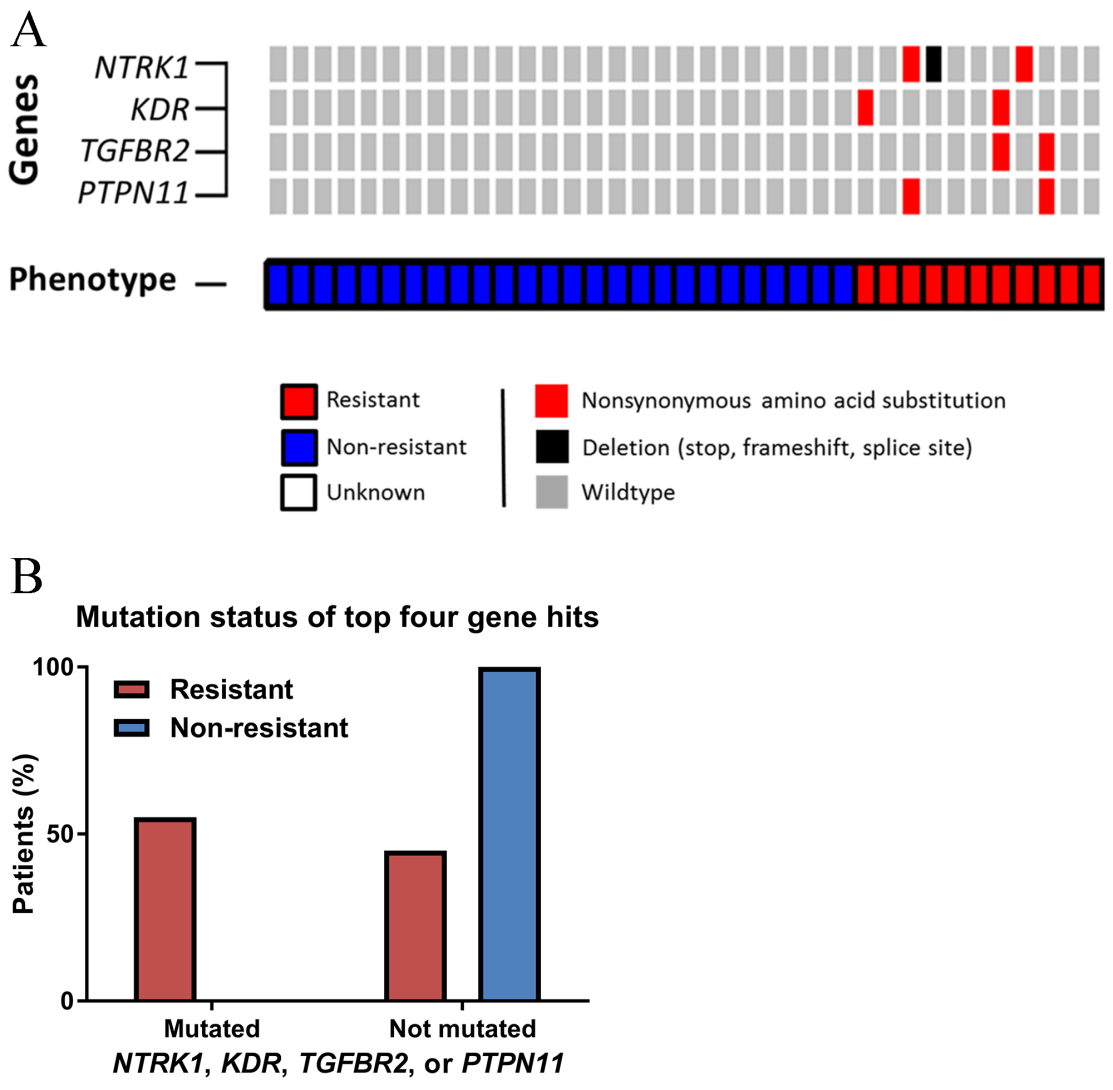
Differences in next-generation sequencing variants **(A)** OncoPrint of somatic nonsynonymous point mutations and insertions/deletions observed differentially by phenotype. **(B)** Distribution of somatic nonsynonymous point mutations or splice site variants in the four key genes.

We identified four genes (*NTRK1*, *KDR*, *TGFBR2*, and *PTPN11*) more commonly mutated in resistant patients than non-resistant patients (Figure 2A). The finding with the lowest p-value was *NTRK1*, in which 3 (30%) resistant patients carried somatic coding mutations (2 point mutations and 1 splice site variant) versus zero non-resistant patients (p = 0.02). Nonsynonymous coding mutations in *KDR, PTPN11,* and *TGFBR2* were present in two (20%) resistant patients and zero non-resistant patients (p = 0.08). Altogether, 55% of resistant patients harbored mutations in one or more of these genes, while zero non-resistant patients carried mutations in these genes (Fisher’s exact p-value = 0.0002, Figure 2B). Gene set enrichment identified trends of receptor binding, activity, protein kinase, tyrosine, and transmembrane within the top gene hits. Of the four top hits, *KDR* and *PTPN11* were included in screening 1,001 cancer cell lines in the Genomics in Drug Sensitivity in Cancer database [14, 15]. Presence of *KDR* mutations was consistently predicted to confer resistance to multi-targeted TKIs, while presence of *PTPN11* mutations tended to predict sensitivity to multi-targeted TKIs.

### Copy number alterations

**Figure 3:**
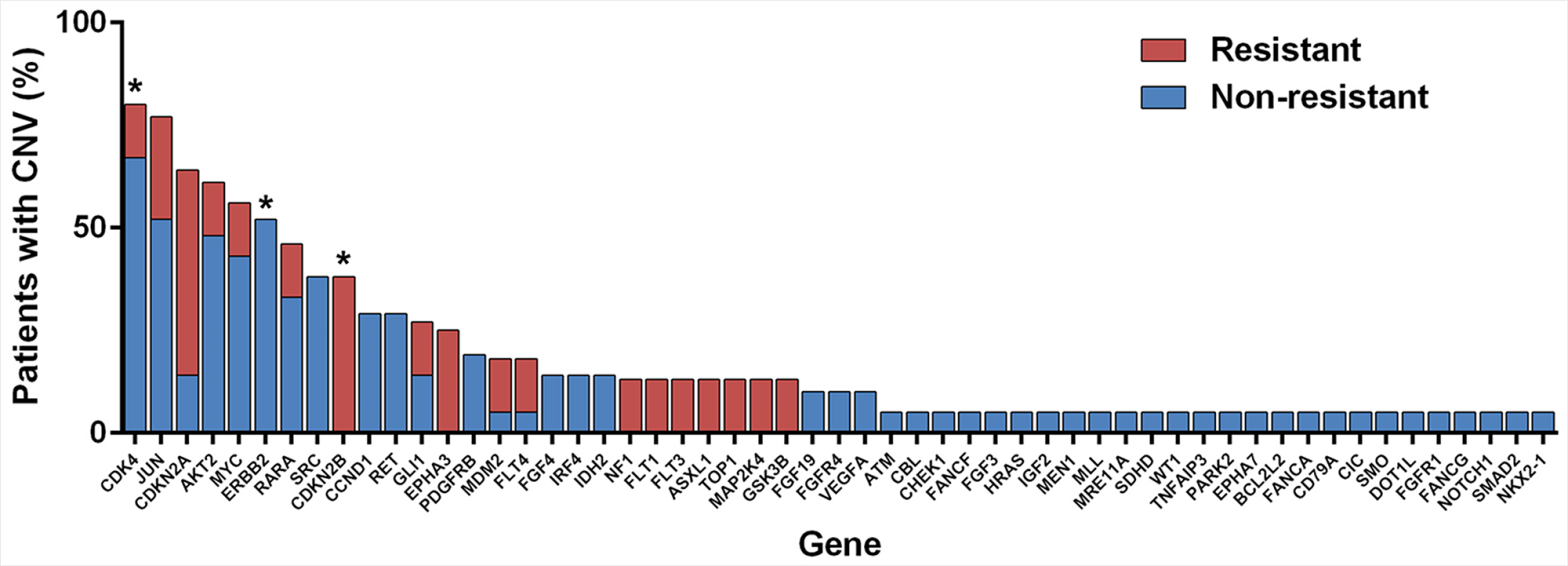
Copy number alterations (CNAs) observed by phenotype ^*^Genes that met pre-specified cut-off for exploratory hits (i.e., differential CNAs between resistant and non-resistant individuals).

Somatic CNA data was generated for 29 individuals, of which 8 (27.6%) were classified as resistant. All samples resulted in data that met pre-specified quality control criteria. Individuals exhibited a diverse range of somatic CNAs, with some individuals demonstrating much more genomic instability than others. A total of 55 (22.6%) genes harbored CNAs that met specified filtering criteria (Figure 3). No genes met the FDR-corrected significance level; however, three genes (*CDK4*, *CDKN2B*, and *ERBB2*) met the exploratory cut-off (FDR-corrected p < 0.3). CNAs in *CDKN2B* were only observed in resistant patients, while CNAs in *CDK4* and *ERBB2* were less common in resistant patients (only non-resistant patients harbored CNAs in *ERBB2*). All of the CNAs in *CDKN2B* and *ERBB2* were homozygous losses, and the majority (14/16, 87.5%) of aberrations in *CDK4* were also losses. Gene set enrichment identified cancer pathways, cyclin, and kinase as network trends between the three gene hits for CNA.

### Decision tree for combined data

**Figure 4:**
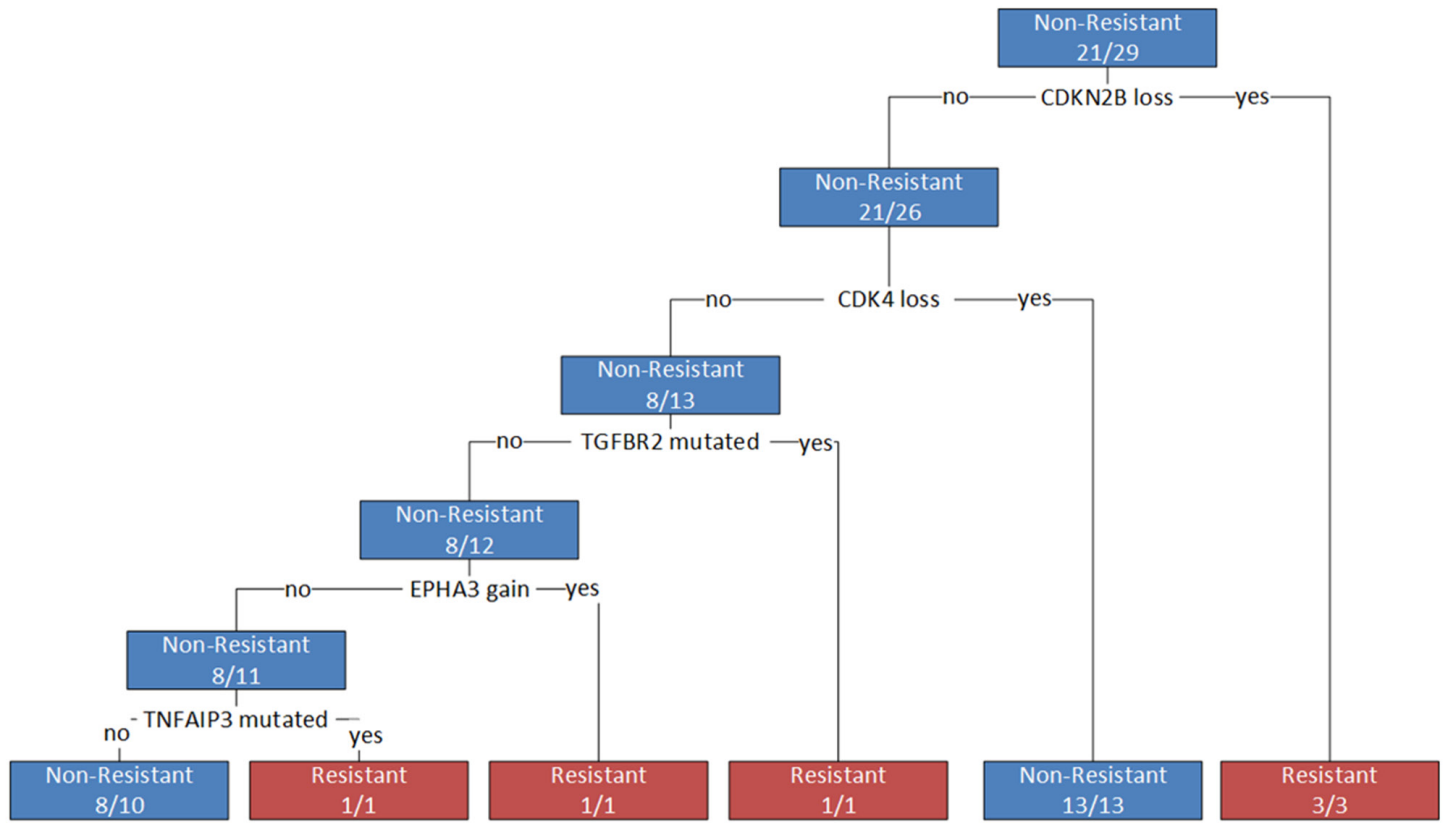
Decision tree for differentiating resistant from non-resistant patients Branches represent decisions based on genes identified as influential in differentiating phenotypes. A loss represents a homozygous copy number loss; a gain represents a copy number gain greater than seven; a mutation represents any non-synonymous or missense mutation in a coding region of the gene.

The most informative CNA and next-generation sequencing features from the random forest classification model were used to generate a decision tree for identifying resistant individuals. After quality control, data for 29 individuals with sequencing and CNA results were included in the construction of the final tree. Five genes (*CDKN2B*, *CDK4*, *TGFBR2*, *EPHA3*, and *TNFAIP3*) were identified as important for differentiating resistant from non-resistant individuals and were selected as features in the final decision tree (Figure 4). CNA in *CDKN2B* and *CDK4* were the most informative and explained responses for 55% (16/29) of the population. Interestingly, in measuring the importance of variables using the mean decrease in Gini score, all gene hits from individual sequencing (*NTRK1*, *KDR*, *TGFBR2*, and *PTPN11*) and CNA (*CDK4*, *CDKN2B*, and *ERBB2*) analysis were identified as being amongst the most informative variables (Figure 4, Supplementary Table 2). The decision tree model resulted in a high sensitivity and specificity for differentiating resistant individuals (0.75 and 1, respectively; balanced accuracy 0.88); however, leave-one-out cross-validation (LOOCV) resulted in a lower sensitivity and specificity (0.25 and 0.95, respectively; balanced accuracy 0.6), indicating that the model constructed on all of the data is overfitting. Although the sensitivity dropped substantially after LOOCV, model specificity remained high, suggesting these features are robustly able to distinguish non-resistant samples in this rather small cohort. It further suggested that having the identified combination of mutations increases one’s odds of being resistant (OR 6.67), however this result was not statistically significant (p>0.05). These features may be good candidates for follow-up analysis, but extensive external validation and larger sample sizes will be needed to develop a more clinically relevant predictive model.

## DISCUSSION

We conducted a retrospective candidate gene study to identify somatic point mutations and copy number alterations associated with intrinsic resistance to multi-targeted TKIs. Using next-generation sequencing, we identified four genes commonly mutated in resistant patients, but not mutated in non-resistant patients: *NTRK1*, *KDR*, *TGFBR2*, and *PTPN11*. Interestingly, three of the four top hits (*TGFR, KDR,* and *NTRK1*) encode transmembrane protein kinases that are known targets of multi-targeted TKIs to varying degrees. The final gene, *PTPN11,* encodes SHP2, a tyrosine phosphatase that mediates signaling of oncogenic tyrosine kinases, such as Ras-ERK-AKT signaling pathways.

All four of the top gene hits from sequencing analysis are known to be mutated in cancer patients and have some literature suggesting possible associations with resistance and/or prognosis. *KDR* encodes the vascular endothelial growth receptor 2 (VEGFR2), a tyrosine kinase that mediates VEGF-induced endothelial proliferation, survival, and migration. *KDR* is commonly mutated across cancer types, and is one of the primary targets of the multi-targeted TKIs, with up to eighty percent of activity being inhibited by TKIs [16]. Therefore, mutations in the gene encoding VEGFR2 represent a plausible mechanism of resistance to these agents. In fact, escape from VEGFR2 signaling dependency has been proposed as a mechanism of acquired resistance to the multi-targeted TKIs [17]. In a recent retrospective analysis of archived renal cell carcinoma patients treated with sunitinib, Stubbs and colleagues found no association between *KDR* expression and overall or progression-free survival, but did not assess mutations [18]. However, a retrospective study of 275 sarcoma patients identify a significant correlation between high VEGFR2 protein expression and decreased patient survival (p <0.001) [19].

The most common *NTRK1* alterations observed in cancer are gene fusions; however, point mutations have also been reported in numerous solid tumors [20, 21]. Multiple studies have linked *NTRK1* overexpression to tumor progression and poor outcomes in solid cancers [22–24], and *NTRK1* mutations confer acquired resistance to NTRK inhibitors [25]. Somatic mutations of *TGFBR2* are commonly observed across solid tumor types [20, 21]. The majority of studies exploring the clinical significance of *TGFBR2* mutations are in the context of breast cancer, where high expression of *TGFBR2* is associated with tumor metastasis and response to chemotherapy [26–28]. Associations between somatic *TGFBR2* alterations and cancer progression occur in a range of solid tumor types, including gastric, bladder, and squamous cell carcinoma [29–31]. *PTPN11* mutations are most commonly associated with Noonan syndrome and juvenile myelomonocytic leukemia; however, activating somatic mutations have been observed in solid tumors, including colorectal, breast, and renal cell carcinomas [20, 21]. These mutations enhanced cancer progression, invasion, and metastasis [32–34] and were associated with decreased response rates in hepatocellular carcinoma, glioma, and gastric cancers [32, 35, 36]. In an elegant *in vitro* study that utilized colon and melanoma cancer cells, Prahallad and colleagues demonstrated that *PTPN11* activating mutations were present in the setting of both intrinsic and acquired resistance and that inhibition of *PTPN11* is lethal in cancer cells driven by activated tyrosine kinases [37].

**Figure 5:**
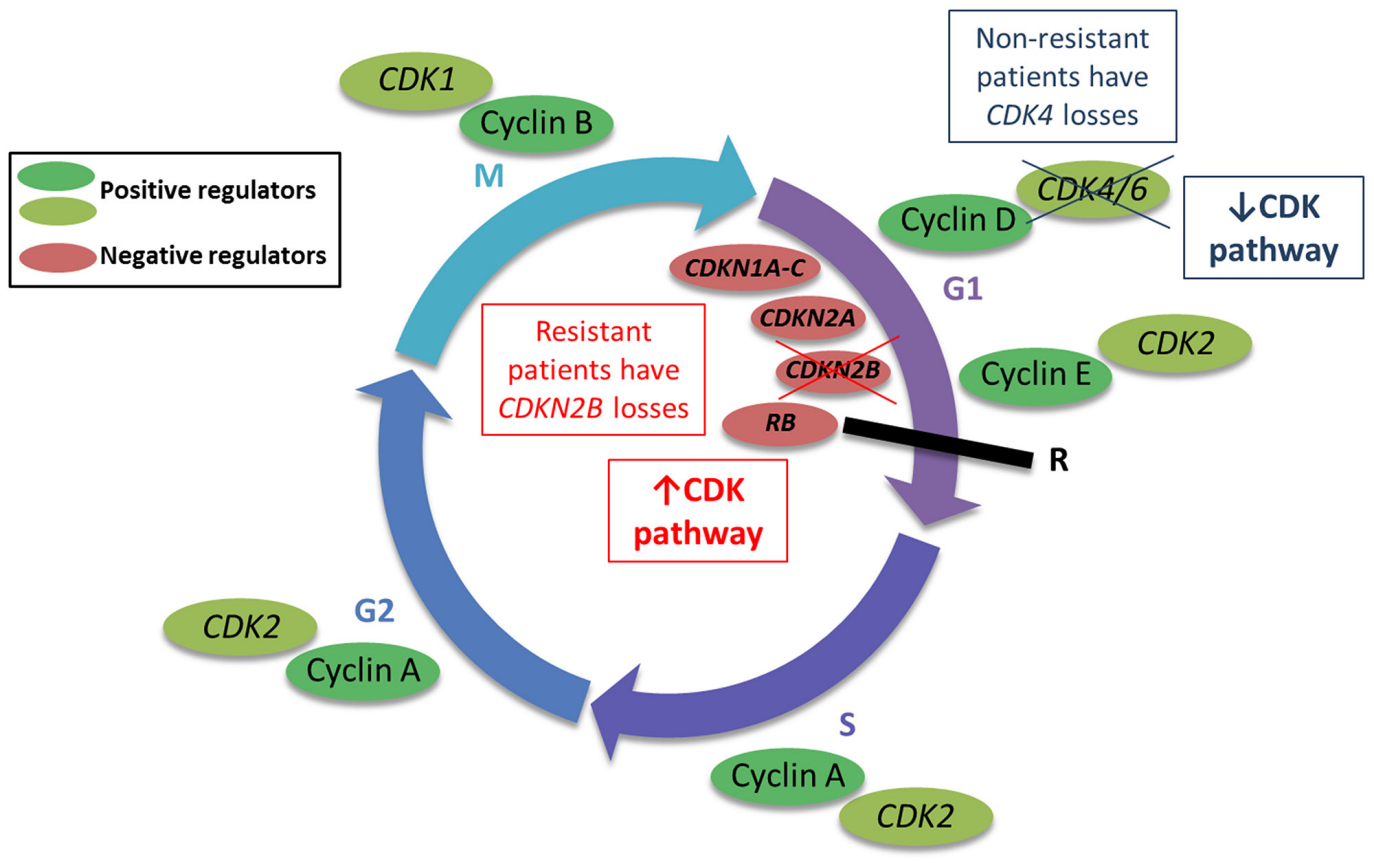
Copy number alterations (CNAs) in the cyclin-dependent (cyclin D) pathway may regulate progression through the cell cycle The cyclin D pathway regulates progression through the cell cycle, ultimately regulating cell division (mitosis, or M phase). The CNAs in genes relevant to this pathway may modulate cell cycle progression as depicted in the figure.

We identified three genes (*CDK4, CDKN2B,* and *ERBB2*) with differential patterns of CNAs between resistant and non-resistant patients. Interestingly, *CDK4* and *CDKN2B* both encode for proteins involved in the cyclin-dependent (cyclin D) pathway, which regulates progression through the cell cycle. The cyclin D pathway is commonly dysregulated in solid malignancies through somatic copy number alterations [38]. In our cohort, we observed a higher frequency of *CDKN2B* losses in resistant patients, while non-resistant patients more commonly harbored losses in *CDK4* (Figure 5). Biologically, CDK4 functions as a positive regulator of the cell cycle, while CDKN2B is a negative regulator. Therefore, loss of *CDK4* results in cell cycle arrest and tumor cell senescence, while loss of *CDKN2B* maintains cell cycle progression and tumor cell growth. The CNAs observed in our cohort suggest that cyclin D regulation may serve as an important secondary or bypass track for cancer progression in individuals treated with multi-targeted TKIs. In fact, reduced expression of CDKN2A (a tumor suppressor protein similar to CDKN2B), has been observed in sarcoma progression and correlated with reduced patient survival [39]. *ERBB2* encodes HER2, a transmembrane tyrosine kinase that regulates the PI3K/AKT pathway upstream of mTOR. Interestingly, after therapeutic failure with multi-targeted TKIs, mTOR inhibitors are recommended as treatment options. We observed that patients non-resistant to multi-targeted TKIs more commonly harbored *ERBB2* losses, which suggests that mTOR may not be overregulated in these individuals, but perhaps is an important mechanism of tumorigenesis in the resistant patients.

The combination of using random forest classification models for variable reduction and constructing a decision tree using the combined next-generation sequencing and CNA results generated a translatable model. The genetic alterations that were identified as being most informative were CNAs in *CDKN2B* and *CDK4*, further supporting a potential roll of the cyclin D pathway in resistance to multi-targeted TKIs. *EPHA3* and *TNFAIP3* were identified as important based on the random forest classification model, but were not identified in separate next-generation sequencing or CNA analyses; however, both of these genes have been previously associated with prognosis and resistance. *EPHA3* encodes a protein tyrosine kinase receptor and its expression has been associated with high invasive capacity and poor overall survival in hepatocellular carcinoma, gastric cancer, and glioblastoma [40–42]. Additionally, *EPHA3* has been associated with the regulation of multi-drug resistance in small cell lung cancer via the PI3K/BMX/STAT3 signaling pathway [43]. *TNFAIP3* encodes a zinc finger protein that serves as a tumor suppressor through its potent inhibition of the NF-κB signaling pathway [44]. *TNFAIP3* has also been associated with regulating drug resistance in multiple solid tumor types [45, 46]. Therefore, dysregulation of these genes represents a biologically plausible mechanism of intrinsic resistance to multi-targeted TKIs.

While the decision tree explained our data well, it appears to be overfitted based on the reduced performance with LOOCV, indicating that additional research is needed to develop a model that will be predictive of patient outcomes. A larger cohort is needed to robustly predict intrinsic resistance in independent datasets. Although this model is not robust enough for clinical use, we believe the five genes identified as being the most important for distinguishing resistant samples are strong candidates for additional follow-up analysis.

The most notable limitation of this study is the sample size. To address this limitation, we elected to use a candidate gene approach to decrease the multiple testing burden. While this was a good method for increasing statistical power, limitations to the candidate gene approach exist, with the main disadvantage being the inability to identify completely novel or unexpected findings. However, the complete paucity of data on resistance to multi-targeted TKIs and, even more-so, intrinsic resistance to multi-targeted TKIs makes any information novel and valuable to this field. Nevertheless, the identification and collection of data from an independent replication cohort would be invaluable to the field. Another limitation of our study, inherent to its retrospective design, was the use of samples collected for clinical purposes as opposed to prospective collection for research purposes. However, the samples included in our study were all collected prior to multi-targeted TKI initiation, with the exception of one. The one individual with a sample collected after TKI treatment was intrinsically resistant to sorafenib, and we believe that any mutations that conferred intrinsic resistance would persist during and after TKI treatment.

We conducted an exploratory study to identify somatic point mutations, insertions/deletions, and copy number alterations characteristic in individuals with intrinsic resistance to multi-targeted TKIs. We identified potential predictors of resistance that each have biological plausibility; however, we acknowledge that multiple other factors may be important in determining who will respond to these agents. For example, pharmacokinetics and pharmacodynamics may affect drug penetration and exposure. Using MALDI-MSI (matrix-assisted laser desorption ionization mass spectrometry imaging) to visualize the distribution patterns of multi-targeted TKIs in mouse models, Torok and colleagues determined that poor drug penetration in some tumors resulted in primary resistance [47]. Clinical variability in drug concentrations, despite receiving the same dose, was associated with variability in side effects and survival outcomes [48, 49]. Lysosomal sequestration has also been demonstrated to confer resistance to the multi-targeted TKIs [50, 51] and was associated with conferring cross-resistance to the multi-targeted TKIs [52]. Finally, germline genetics in drug transporters, such as *ABCB1* and *ABCG2*, or pharmacodynamic proteins, such as *BIM*, may also influence response to these agents [53–55]. While many factors must be considered when developing an optimized algorithm for predicting resistance in clinical practice, this study identified a combination of mutations and CNA that were associated with resistance to multi-targeted TKIs. With additional validation, preemptive identification of patients intrinsically resistant to multi-targeted TKIs will inform personalized treatment decisions to maximize outcomes while avoiding unnecessary exposures and toxicities.

## MATERIALS AND METHODS

### Patient population

Patients were identified from the Total Cancer Care (TCC) cohort at Moffitt Cancer Center, an institutional review board (IRB)-approved biobanking protocol (MCC14690, MCC13579) in which individuals agree to provide tissue and blood samples for research and to be followed throughout their lifetime [56]. Additional Moffitt Cancer Center Scientific Review Committee and IRB-approvals were granted to access tissue samples and conduct this particular study (MCC18790). Individuals eligible for study inclusion were patients consented between January 1, 1994 and December 31, 2015 over the age of 18 years diagnosed with any type of solid tumor and treated with a multi-targeted TKI (axitinib, cabozantinib, pazopanib, regorafenib, sorafenib, sunitinib, or vandetanib). Individuals were included if they had targeted exome sequencing or whole exome sequencing (WES) data available through the TCC protocol or if a tumor FFPE sample was available that could be used for DNA extraction and WES. Individuals treated with a multi-targeted TKI for a hematologic malignancy were excluded.

### Phenotyping

Data was initially abstracted in a standardized manner from the TCC biorepository system (TransMed, Cupertino, CA). Data abstracted included demographic variables (date of birth, gender, race, and ethnicity) as well as clinical information [medical record numbers (MRNs), date of diagnosis, primary site at diagnosis, histology, first course of treatment, multi-targeted TKI received, start and stop date of TKI, and date of death or last follow-up]. Sources for these data were the Florida Cancer Registry and electronic medical records. Information abstracted from TransMed was used to guide manual chart reviews.

Two clinicians and researchers performed independent manual chart reviews using the patients’ MRNs. Chart reviews included the validation of information generated using TransMed abstraction and manual review of patients’ clinical notes and imaging (PET/CT/MRI) results before and after TKI administration. Data collected included radiologists’ and clinicians’ impressions of disease before and after TKI initiation (e.g., stable vs. progressive vs. responding, based on imaging studies) and time to drug change or discontinuation. Physicians’ recommendations at first imaging follow-up (generally 2-3 months post-initiation) were used to identify patients intrinsically resistant to multi-targeted TKIs. The decision to stop TKI therapy due to cancer progression on imaging studies was classified as intrinsic resistance. Patients who continued TKI therapy due to response, stable disease, or mixed response on first imaging follow-up, and patients who met these criteria but stopped therapy due to side effects were classified as non-resistant. Individuals who stopped therapy early due to side effects or were lost to follow-up (i.e., those without first-imaging follow-up) could not confidently be classified were excluded from analyses. Based on these data, reviewers documented their individual impressions of patients as either resistant or non-resistant. Phenotype classifications were then compared and a third reviewer, a medical oncologist experienced with TKI prescribing and follow-up, provided adjudication when necessary.

### Next-generation sequencing and copy number alterations

WES was performed in order to identify somatic mutations in the coding regions of the human genome. Briefly, 200 ng of DNA, as quantified by qPCR, was used as input for library preparation with the SureSelect^XT^ Reagent Kit (Agilent, Santa Clara, CA). For each tumor DNA sample, a genomic DNA library was constructed according to the SureSelect^XT^ Target Enrichment for Illumina Multiplexed Sequencing (#G7530-90000) protocol (Agilent), including the suggested modifications for FFPE-derived DNA samples. The pre-captured library was amplified, and the size and quality of the library was evaluated using a 2100 BioAnalzyer (Agilent) and Qubit™ quantification (Thermo Fisher Scientific, Waltham, MA). Approximately 500 to 750 ng of pre-captured library was used for hybridization at 65°C for 24 hr. Hybridization and target enrichment were conducted using the SureSelect^XT^ Clinical Research Exome kit (Agilent). The post-captured library was amplified and evaluated with a 2100 Bioanalyzer (Agilent). The enriched library was quantified using a Library Quantification Kit for NGS (KAPA Biosystems, Wilmington, MA), and samples were diluted to a 4 nM concentration. Denaturation was conducted using NaOH, followed by neutralization with Tris buffer pH 8.5, and samples were diluted to a concentration of 20 pM in HT1. Next, samples were diluted to concentrations between 1.7 pM to 2.2 pM for sequencing with a v2 sequencing reagent kit and a NextSeq 500 desktop sequencer (Illumina, San Diego, CA). Approximately 85 million 75 base paired-end reads were generated for each DNA sample.

Individuals with targeted gene sequencing data available had sequencing performed previously through collaboration with the Beijing Genomics Institute (Shenzhen, China). Briefly, tumor samples underwent targeted gene sequencing using a custom SureSelect platform (Agilent, Santa Clara, CA) targeting 1,321 cancer-related genes and 2 × 90bp massively parallel sequencing using a Genome Analyzer IIx (Illumina, San Diego, CA). In order to identify whole genome CNA and loss-of-heterozygosity, the OncoScan^®^ FFPE Assay Kit (Affymetrix, Santa Clara, CA) was used according to the manufacturer’s protocol, with an input of 80 ng of FFPE-extracted DNA.

### Candidate gene selection

To decrease multiple comparison correction and increase the likelihood of identifying biologically plausible associations, we utilized a candidate gene approach for analysis. Candidate genes included genes known to be important in solid tumor biology and anticancer drug response were selected utilizing the overlapping genes reported on clinical cancer genetic testing platforms (such as FoundationOne^®^) and those captured with the targeted sequencing platform designed specifically for the TCC study cohort (Supplementary Table 1).

### Data quality control and variant detection

Whole exome sequencing reads were aligned to the reference human genome (hs37d5) with the Burrows-Wheeler Aligner [57], and duplicate identification, insertion/deletion realignment, quality score recalibration, and variant identification were performed with Picard (Broad Institute, http://broadinstitute.github.io/picard/) and the Genome Analysis ToolKit (GATK) v2.2-Lite [58]. Genotypes were determined across all samples at variant positions. Sequence variants were annotated to determine genic context (i.e., non-synonymous, missense, splicing) using ANNOVAR [59], and summarized using spreadsheets and a genomic data visualization tool, VarSifter [60]. Additional contextual information from other studies was added, including allele frequency from 1000 Genomes [61] and the NHLBI Exome Sequence Project [62], *in silico* function impact predictions (PolyPhen and SIFT), and observed impacts from databases including ClinVar (NCBI, http://www.ncbi.nlm.nih.gov/clinvar/), the Catalogue of Somatic Mutations in Cancer (COSMIC) [63], and The Cancer Genome Atlas (TCGA, https://cancergenome.nih.gov/). Somatic mutations were prioritized by excluding variants observed in 1000 Genomes [61] and variants observed at >5% in an internal dataset of adjacent normal (i.e., non-tumor) tissue. Variants with GATK variant quality score recalibration (VQSR) tranche > 99.9 or genotype quality (GQ) < 15 were excluded.

The CNA data generated was analyzed using Nexus Copy Number™ 6.0 software (BioDiscovery, El Segundo, CA) utilizing the TuScan™ algorithm, specifically designed for OncoScan^®^ FFPE Assay data [64]. The estimated CNA regions were annotated with the reference human genome (hg19) and the evaluation of array performance was measured using default criteria (MAPD ≤ 0.3 and ndSNPQC ≥ 26). Plots of whole genome CNAs and minor (or B) allele frequencies (BAFs) were generated for each individual. Briefly, the BAF was calculated as the count of minor (B) alleles (A/T) divided by the total count of major (A) (G/C) and minor (B) alleles [65]. Using the TuScan™ algorithm the average CNA of all cells within each sample was generated. Genes with 70% or greater overlap in copy number aberrant regions were classified as being altered. Copy number gains greater than seven and homozygous losses were considered potentially clinically significant, as is standard in clinical tumor testing, and included in analyses.

### Data analysis

Patients were classified into one of two cohorts: (1) resistant or (2) non-resistant using the phenotyping methods described above. Descriptive statistics were used to summarize demographic and clinical characteristics of patients included. Means, standard deviations and ranges were calculated for continuous variables, and frequencies and percentages were generated for categorical variables. As a conservative approach and where applicable, non-parametric statistical tests were implemented to avoid assuming the data was normally distributed. Demographic and clinical characteristic comparisons between resistant and non-resistant patients were performed using two-tailed Fisher’s exact tests for categorical variables and the Mann-Whitney U test for continuous variables. Batch effects between the two sequencing methods and differences between tumor types were detected using a two-tailed Fisher’s exact test and, when comparing between sequencing platforms, mutated genes with an FDR-corrected p-value < 0.05 were excluded from further analyses. The two-tailed Fisher’s exact test was used to test the statistical significance of mutated candidate genes identified in resistant and non-resistant patients. An FDR-corrected p-value < 0.1 was considered statistically significant, and an uncorrected p-value < 0.1 was significant for exploratory associations. Cytoscape (v.3.4.0) Enrichment Map [66] was utilized to conduct gene set enrichment of top gene hits, and the Genomics in Drug Sensitivity in Cancer database [67] was utilized to explore *in vivo-*derived predictions of sensitivity to multi-targeted TKIs based on mutation status of the top hits.

For CNA analysis, copy number gains and losses were grouped. Presence of copy number aberrations between resistant and non-resistant individuals were arranged in contingency tables and analyzed using a two-sided Fisher’s exact test. FDR-corrected p-values < 0.1 were considered statistically significant, and a threshold of FDR-corrected p < 0.3 was used for exploratory analysis [68, 69]. Cytoscape (v.3.4.0) Enrichment Map [66] was utilized to conduct gene set enrichment of top gene hits.

Statistical analysis was conducted using the open-source, statistical programming language, R (version 3.3.2) [70]. CNA and WES data were combined to build a model leveraging both technologies. CNAs were annotated as 1, 0, −1 for gain, no change, or loss, respectively, based on the criteria described above. Subjects with >20% missing data and genes with >5% missing data were excluded from further analysis. Remaining missing genes were imputed using the mean of the study cohort. Subsequently, feature reduction, via a random forest model was constructed using 2000 trees and 12 features at each split [71]. CNA and WES features with a mean decrease in Gini score >0.2, as determined using the elbow method, were selected for inclusion in the decision tree classification model using recursive partitioning trees [72]. Figures were generated using R (version 3.3.2) and GraphPad Prism 6.

## SUPPLEMENTARY MATERIALS TABLES





## Abbreviations

BAF: minor (or B) allele frequencies
CNA: copy number alteration
CT: computed tomography
cyclin D: cyclin-dependent
EGFR: epidermal growth factor receptor
FDA: United States Food and Drug Administration
FDR: false discovery rate
FFPE: formalin-fixed paraffin-embedded
GATK: Genome Analysis ToolKit
GQ: genotype quality
IRB: institutional review board
MALDI-MSI: matrix-assisted laser desorption ionization mass spectrometry imaging
MRI: magnetic resonance imaging
MRN: medical record numbers
PET: positron emission tomography
TCC: Total Cancer Care
TCGA: The Cancer Genome Atlas
TKIs: tyrosine kinase inhibitor
VQSR: variant quality score recalibration
WES: whole exome sequencing.
