# Supplementary material for: Tumor exome sequencing and copy number alterations reveal potential predictors of intrinsic resistance to multi-targeted tyrosine kinase inhibitors
Source: Oncotarget. 2017 Dec 4;8(70):115114–27. doi: 10.18632/oncotarget.22914 (PMC5777758; doi:10.18632/oncotarget.22914)
Supplement: Supplementary file 1 [file oncotarget-08-115114-s001.pdf]

Tumor exome sequencing and copy number alterations reveal potential predictors of intrinsic resistance to multi-targeted tyrosine kinase inhibitors

SUPPLEMENTARY MATERIALS

Supplementary Table 1: Candidate genes (n = 243) included in next generation sequencing and copy number alteration analyses

ABL1	ABL2	ACVR1B	AKT1	AKT2	AKT3	ALK	APC
AR	ARAF	ARFRP1	ARID1A	ASXL1	ATM	ATR	ATRX
AURKA	AURKB	AXIN1	AXL	BAP1	BARD1	BCL2	BCL2L1
BCL2L2	BCL6	BCORL1	BLM	BRAF	BRCA1	BRCA2	BRIP1
BTK	CARD11	CBL	CCND1	CCND2	CCND3	CCNE1	CD79A
CD79B	CDC73	CDH1	CDK12	CDK4	CDK6	CDK8	CDKN1A
CDKN1B	CDKN2A	CDKN2B	CDKN2C	CEBPA	CHEK1	CHEK2	CIC
CREBBP	CRKL	CRLF2	CSF1R	CTNNA1	CTNNB1	CYLD	DAXX
DDR2	DNMT3A	DOT1L	EGFR	EP300	EPHA3	EPHA5	EPHA7
EPHB1	ERBB2	ERBB3	ERBB4	ERG	ERRFI1	ESR1	EZH2
FANCA	FANCC	FANCD2	FANCE	FANCF	FANCG	FANCL	FAS
FBXW7	FGF10	FGF19	FGF3	FGF4	FGFR1	FGFR2	FGFR3
FGFR4	FH	FLT1	FLT3	FLT4	FOXL2	GATA1	GLI1
GNA11	GNAQ	GNAS	GRIN2A	GRM3	GSK3B	HGF	HNF1A
HRAS	HSP90	IDH1	IDH2	IGF1R	IGF2	IKBKE	IKZF1
INHBA	INPP4B	IRF4	IRS2	JAK1	JAK2	JAK3	JUN
KDM5A	KDM5C	KDM6A	KDR	KEAP1	KIT	KRAS	LRP1B
LYN	MAGI2	MAP2K1	MAP2K2	MAP2K4	MAP3K1	MCL1	MDM2
MDM4	MEN1	MET	MITF	MLH1	MLL	MLL2	MLL3
MPL	MRE11A	MSH2	MSH6	MTOR	MUTYH	MYC	MYCN
MYD88	NF1	NF2	NKX2-1	NOTCH1	NOTCH2	NOTCH3	NPM1
NRAS	NTRK1	NTRK2	NTRK3	PAK3	PALB2	PARK2	PAX5
PDGFRA	PDGFRB	PDK1	PIK3C2B	PIK3CA	PIK3CB	PIK3CG	PIK3R1
PIK3R2	PLCG2	PMS2	POLE	PPP2R1A	PREX2	PRKAR1A	PRKCI
PRKDC	PTCH1	PTEN	PTPN11	RAC1	RAD50	RAD51	RAF1
RARA	RB1	RET	RICTOR	ROS1	RPTOR	RUNX1	RUNX1T1
SDHB	SDHC	SDHD	SETD2	SMAD2	SMAD3	SMAD4	SMARCA4
SMARCB1	SMO	SOCS1	SOX10	SOX2	SPEN	SPOP	SRC
STAT3	STAT4	STK11	SUFU	SYK	TERT	TET2	TGFBR2
TNFAIP3	TOP1	TOP2A	TP53	TSC1	TSC2	TSHR	VEGFA
VHL	WT1	ZNF217					

Supplementary Table 2: Mean decrease GINI scores for decision tree analysis. Values for non-resistant and resistant represent the mean raw importance score for the alteration for classification of that genotype. The Mean decrease accuracy represents the proportion of observations that are incorrectly classified by removing the alteration. The Mean decrease Gini score represents the number of splits (across all trees) that include that alteration, proportionally to the number of samples it splits. Therefore, the larger the decrease Gini score, the more important that alteration is in differentiating resistant from non-resistant individuals. Alterations with a mean decrease Gini > 0.2 were included as possible features in the decision tree classification model (highlighted yellow). Bolded alterations are those features that were ultimately selected in the final decision tree model. Remaining factors with a mean decrease Gini > 0.2 that are not in the decision tree are still considered to be important, but do not add enough additional information to those selected for inclusion in the model.

See Supplementary File 1
