# Supplementary material for: Tumor exome sequencing and copy number alterations reveal potential predictors of intrinsic resistance to multi-targeted tyrosine kinase inhibitors
Source: Oncotarget. 2017 Dec 4;8(70):115114–27. doi: 10.18632/oncotarget.22914 (PMC5777758; doi:10.18632/oncotarget.22914)
Supplement: Supplementary file 2 [file oncotarget-08-115114-s002.doc]

**Supplementary Table 2: Mean decrease GINI scores for decision tree analysis**

Values for non-resistant and resistant represent the mean raw importance score for the alteration for classification of that genotype. The mean decrease accuracy represents the proportion of observations that are incorrectly classified by removing the alteration. The mean decrease Gini score represents the number of splits (across all trees) that include that alteration, proportionally to the number of samples it splits. Therefore, the larger the decrease Gini score, the more important that alteration is in differentiating resistant from non-resistant individuals. Alterations with a mean decrease Gini > 0.2 were included as possible features in the decision tree classification model (highlighted yellow). Bolded alterations are those features that were ultimately selected in the final decision tree model. Remaining factors with a mean decrease Gini > 0.2 that are not in the decision tree are still considered to be important, but do not add enough additional information to those selected for inclusion in the model.

|  | **Non-resistant** | **Resistant** | **Mean decrease accuracy** | **Mean decrease Gini score** |
| --- | --- | --- | --- | --- |
| **cnv_CDKN2B** | **0.0106** | **0.0255** | **0.0145** | **0.6324** |
| **wes_TGFBR2** | **0.0040** | **0.0073** | **0.0045** | **0.3909** |
| **cnv_EPHA3** | **0.0039** | **0.0070** | **0.0047** | **0.3764** |
| **cnv_CDK4** | **0.0004** | **0.0293** | **0.0070** | **0.3686** |
| **cnv_ERBB2** | **0.0038** | **0.0264** | **0.0084** | **0.3444** |
| wes_KDR | 0.0025 | 0.0054 | 0.0032 | 0.3405 |
| wes_PTPN11 | 0.0039 | 0.0067 | 0.0045 | 0.3310 |
| cnv_CDKN2A | 0.0014 | 0.0065 | 0.0026 | 0.3009 |
| **wes_TNFAIP3** | **0** | **0** | **0** | **0.2865** |
| wes_NTRK1 | 0.0024 | 0.0045 | 0.0029 | 0.2431 |
| wes_RICTOR | -0.0077 | -0.0094 | -0.0074 | 0.2257 |
| wes_TERT | 0.0010 | 0.0016 | 0.0011 | 0.2182 |
| wes_SDHD | 0 | 0 | 0 | 0.1691 |
| wes_PRKDC | -0.0006 | -0.0022 | -0.0009 | 0.1689 |
| wes_IKZF1 | 0.0021 | -0.0028 | 0.0006 | 0.1681 |
| cnv_SRC | -0.0007 | 0.0094 | 0.0017 | 0.1604 |
| wes_APC | -0.0014 | -0.0022 | -0.0016 | 0.1521 |
| wes_AR | -0.0013 | -0.0062 | -0.0022 | 0.1493 |
| cnv_JUN | -0.0041 | 0.0060 | -0.0016 | 0.1411 |
| cnv_AKT2 | -8.91E-06 | 0.0071 | 0.0016 | 0.1369 |
| wes_IKBKE | -0.0011 | -0.0023 | -0.0014 | 0.1345 |
| wes_VHL | 0.0004 | 0.0070 | 0.0018 | 0.1324 |
| cnv_MYC | -0.0034 | 0.0029 | -0.0019 | 0.1234 |
| cnv_TOP1 | 0 | 0 | 0 | 0.1233 |
| wes_NOTCH2 | -0.0007 | -0.0032 | -0.0015 | 0.1229 |
| wes_JAK2 | 0.0024 | 0.0049 | 0.0030 | 0.1156 |
| wes_AXIN1 | -0.0011 | -0.0026 | -0.0014 | 0.1120 |
| cnv_FLT1 | 0 | 0 | 0 | 0.1120 |
| wes_NRAS | 0 | 0 | 0 | 0.1119 |
| cnv_FLT3 | 0 | 0 | 0 | 0.1100 |
| cnv_GSK3B | 0 | 0 | 0 | 0.1091 |
| cnv_NF1 | 0 | 0 | 0 | 0.1089 |
| cnv_ASXL1 | 0 | 0 | 0 | 0.1053 |
| cnv_MAP2K4 | 0 | 0 | 0 | 0.1048 |
| wes_CBL | 0 | 0 | 0 | 0.1047 |
| wes_JAK3 | -0.0005 | -0.0038 | -0.0013 | 0.1035 |
| wes_NOTCH1 | 0 | 0 | 0 | 0.1032 |
| wes_FOXL2 | -0.0002 | 0.0005 | -0.0001 | 0.0969 |
| wes_ATR | -0.0004 | 0.0039 | 0.0006 | 0.0949 |
| cnv_CCND1 | -0.0013 | 0.0056 | 0.0001 | 0.0941 |
| wes_PMS2 | -0.0014 | -0.0019 | -0.0014 | 0.0941 |
| wes_MLL | -0.0007 | -0.0011 | -0.0008 | 0.0937 |
| wes_DAXX | 0 | 0 | 0 | 0.0918 |
| wes_STAT4 | 0 | 0 | 0 | 0.0882 |
| wes_PIK3C2B | 0 | 0 | 0 | 0.0878 |
| wes_FLT4 | -0.0005 | -0.0031 | -0.0010 | 0.0850 |
| wes_BRCA2 | -7.72E-05 | -0.0024 | -0.0006 | 0.0843 |
| wes_MLL3 | -0.0022 | 0.0015 | -0.0013 | 0.0829 |
| wes_BARD1 | 0.0002 | -0.0009 | -4.66E-05 | 0.0820 |
| wes_ATRX | 0 | 0 | 0 | 0.0813 |
| wes_TP53 | -2.52E-05 | -0.0023 | -0.0006 | 0.0744 |
| cnv_RARA | -0.0019 | 0.0010 | -0.0010 | 0.0713 |
| cnv_RET | -0.0010 | 0.0041 | 9.75E-06 | 0.0667 |
| wes_FLT1 | -0.0006 | -0.0014 | -0.0008 | 0.0665 |
| wes_MDM2 | -0.0006 | -0.0007 | -0.0007 | 0.0566 |
| wes_BAP1 | 0.0005 | 0.0008 | 0.0006 | 0.0561 |
| cnv_FLT4 | -0.0004 | -0.0007 | -0.0005 | 0.0545 |
| wes_POLE | 0.0005 | 0.0013 | 0.0006 | 0.0544 |
| wes_EPHA5 | -0.0002 | 0.0008 | 1.13E-05 | 0.0540 |
| wes_CDH1 | -0.0003 | -0.0007 | -0.0003 | 0.0508 |
| cnv_MDM2 | -0.0004 | -0.0005 | -0.0004 | 0.0483 |
| wes_CIC | -0.0008 | -0.0021 | -0.0010 | 0.0472 |
| wes_SETD2 | -0.0012 | -8.33E-05 | -0.0009 | 0.0464 |
| wes_PTCH1 | 0 | 0 | 0 | 0.0461 |
| wes_KIT | 0 | 0 | 0 | 0.0411 |
| wes_PDGFRB | 0 | 0 | 0 | 0.0410 |
| wes_MLH1 | 0 | 0 | 0 | 0.0384 |
| wes_PREX2 | -0.0002 | 0.0001 | -0.0001 | 0.0355 |
| wes_TSC1 | -0.0002 | 0.0004 | -5.32E-05 | 0.0345 |
| wes_KDM5C | -0.0001 | 0.0010 | 0.0001 | 0.0344 |
| wes_SDHC | 0 | 0 | 0 | 0.0300 |
| wes_JUN | 0 | 0 | 0 | 0.0296 |
| wes_KDM5A | -0.0006 | -0.0005 | -0.0005 | 0.0296 |
| cnv_GLI1 | -0.0006 | -0.0016 | -0.0007 | 0.0286 |
| wes_CDKN1B | 0 | 0 | 0 | 0.0268 |
| wes_RAF1 | 0 | 0 | 0 | 0.0257 |
| wes_SMO | 0 | 0 | 0 | 0.0253 |
| wes_HNF1A | 0 | 0 | 0 | 0.0232 |
| wes_TOP2A | 0 | 0 | 0 | 0.0218 |
| wes_NTRK2 | 0 | 0 | 0 | 0.0218 |
| wes_FANCL | 0 | 0 | 0 | 0.0215 |
| cnv_PARK2 | 0 | 0 | 0 | 0.0207 |
| cnv_PDGFRB | -0.0004 | 0.0011 | -8.47E-05 | 0.0202 |
| wes_ROS1 | 0 | 0 | 0 | 0.0198 |
| wes_FGFR3 | 0 | 0 | 0 | 0.0198 |
| cnv_EPHA7 | 0 | 0 | 0 | 0.0198 |
| wes_ALK | 0 | 0 | 0 | 0.0195 |
| cnv_FGF4 | -6.81E-05 | 0.0005 | 8.26E-05 | 0.0190 |
| wes_EGFR | 0 | 0 | 0 | 0.0182 |
| cnv_TNFAIP3 | 0 | 0 | 0 | 0.0176 |
| wes_AKT1 | 0 | 0 | 0 | 0.0174 |
| wes_BCL6 | 0 | 0 | 0 | 0.0161 |
| wes_BCORL1 | -0.0002 | 0.0009 | -1.41E-05 | 0.0158 |
| cnv_IDH2 | -8.51E-05 | 0.0003 | 2.59E-05 | 0.0158 |
| wes_FH | -0.0005 | 0.0001 | -0.0003 | 0.0142 |
| wes_FANCG | 0 | 0 | 0 | 0.0141 |
| wes_FANCA | 0 | 0 | 0 | 0.0137 |
| cnv_FGF19 | 0 | 0 | 0 | 0.0136 |
| wes_PDGFRA | 0 | 0 | 0 | 0.0136 |
| cnv_IGF2 | 0 | 0 | 0 | 0.0133 |
| cnv_IRF4 | -8.33E-05 | 0.0005 | -2.40E-05 | 0.0130 |
| cnv_WT1 | 0 | 0 | 0 | 0.0122 |
| wes_BRIP1 | 0 | 0 | 0 | 0.0120 |
| wes_ERBB3 | 0 | 0 | 0 | 0.0117 |
| cnv_VEGFA | -6.25E-05 | 0 | -5.00E-05 | 0.0099 |
| cnv_ATM | 0 | 0 | 0 | 0.0098 |
| cnv_MRE11A | 0 | 0 | 0 | 0.0093 |
| wes_RB1 | -0.0001 | 0 | -0.0001 | 0.0092 |
| cnv_CHEK1 | 0 | 0 | 0 | 0.0091 |
| cnv_CBL | 0 | 0 | 0 | 0.0089 |
| wes_BRCA1 | 0 | 0 | 0 | 0.0089 |
| cnv_FGF3 | 0 | 0 | 0 | 0.0088 |
| wes_CHEK2 | 0 | 0 | 0 | 0.0085 |
| wes_AXL | 0 | 0 | 0 | 0.0082 |
| wes_ERBB4 | 0 | 0 | 0 | 0.0082 |
| cnv_SDHD | 0 | 0 | 0 | 0.0078 |
| cnv_MEN1 | 0 | 0 | 0 | 0.0077 |
| cnv_FANCF | 0 | 0 | 0 | 0.0066 |
| wes_FLT3 | 0 | 0 | 0 | 0.0063 |
| wes_SPEN | 0 | 0 | 0 | 0.0062 |
| cnv_HRAS | 0 | 0 | 0 | 0.0062 |
| cnv_FANCA | 0 | 0 | 0 | 0.0057 |
| wes_RPTOR | 0 | 0 | 0 | 0.0053 |
| cnv_MLL | 0 | 0 | 0 | 0.0053 |
| wes_GNAS | 0 | 0 | 0 | 0.0052 |
| wes_ATM | 0 | 0 | 0 | 0.0046 |
| wes_MTOR | 0 | 0 | 0 | 0.0042 |
| wes_ERBB2 | 0 | 0 | 0 | 0.0040 |
| wes_RAD50 | 0 | 0 | 0 | 0.0039 |
| wes_CDKN1A | 0 | 0 | 0 | 0.0038 |
| cnv_SMAD2 | 0 | 0 | 0 | 0.0038 |
| wes_STK11 | 0 | 0 | 0 | 0.0038 |
| cnv_CIC | 0 | 0 | 0 | 0.0038 |
| cnv_NKX2.1 | 0 | 0 | 0 | 0.0036 |
| wes_EP300 | 0 | 0 | 0 | 0.0036 |
| wes_PLCG2 | 0 | 0 | 0 | 0.0033 |
| wes_SMAD4 | 0 | 0 | 0 | 0.0033 |
| wes_TSC2 | 0 | 0 | 0 | 0.0033 |
| wes_NOTCH3 | 0 | 0 | 0 | 0.0030 |
| wes_PIK3CG | 0 | 0 | 0 | 0.0030 |
| cnv_BCL2L2 | 0 | 0 | 0 | 0.0029 |
| wes_CDKN2A | 0 | 0 | 0 | 0.0029 |
| cnv_NOTCH1 | 0 | 0 | 0 | 0.0028 |
| wes_GRIN2A | 0 | 0 | 0 | 0.0024 |
| cnv_SMO | 0 | 0 | 0 | 0.0023 |
| cnv_FGFR1 | 0 | 0 | 0 | 0.0023 |
| cnv_FGFR4 | 0 | 0 | 0 | 0.0020 |
| cnv_CD79A | 0 | 0 | 0 | 0.0020 |
| wes_CTNNA1 | 0 | 0 | 0 | 0.0013 |
| cnv_DOT1L | 0 | 0 | 0 | 0.0006 |
| wes_SMARCA4 | 0 | 0 | 0 | 0.0006 |
| cnv_FANCG | 0 | 0 | 0 | 0.0002 |
